# Knollenblätterpilzvergiftung mit akutem Leberversagen

**DOI:** 10.1007/s00104-025-02330-y

**Published:** 2025-07-21

**Authors:** Maren Schulze, Dieter Hoyer, Simone Kathemann, Elke Lainka, Denisa Pilic, Kristina Kampmann, Matthias Hartmann, Silvio Nadalin, Laura Masilescu, Lars Pape, Ulf Neumann

**Affiliations:** 1 Klinik für Allgemeine, Viszeral‑, Gefäß- und Transplantationschirurgie, Universitätsklinikum Essen, Hufelandstr. 55, 45147 Essen, Deutschland; 2 Klinik für Pädiatrie II, Universitätsklinikum Essen, Essen, Deutschland; 3 Klinik für Anästhesiologie und Intensivmedizin, Essen, Deutschland; 4 Klinik Allgemeine, Viszeral- und Transplantationschirurgie, Universitätsklinikum Tübingen, Tübingen, Deutschland

## Anamnese

Bei unserem Empfänger handelt es sich um einen 12-jährigen Jungen, mit einem Körpergewicht von 46 kg und 152 cm Körpergröße. Das Kind hatte mit seiner Familie Knollenblätterpilze (*Amanita*) gegessen und wurde mit einem akuten Leberversagen in unsere Klinik verlegt. Die Verlegung erfolgte 3 Tage nach Ingestion. Bei Aufnahme hatte der Patient abdominelle Beschwerden, war jedoch orientiert und hatte keine Zeichen einer Enzephalopathie.

## Aufnahmelabor

GOT 3050 U/l, GPT 3491 U/l, Bilirubin 17,1 µl/l, LDH 3073 U/l und Quick 32 %, INR 2,03. Es erfolgte eine sofortige Listung des Patienten, der High-Urgency-Antrag wurde noch am gleichen Tag genehmigt. Zur Plasmapherese wurde in der Nacht ein Hämodialysekatheter gelegt, zu diesem Zeitpunkt betrug der Quick-Wert nur noch 12 %, die maximalen Transaminasen betrugen GOT 5612 U/l, GOT 9049 U/l. Der High-Urgency-Antrag wurde Eurotransplant genehmigt.

## Verlauf

Der Patient wurde täglich einer Plasmapherese unterzogen. Nach 2 Tagen zeigte sich der Patient deutlich agitierter, Ammoniak im Serum war trotz Plasmapherese ansteigend und betrug 363 µg/dl, Laktat maximal 6,60 mmol/l. Die Aufklärung der Eltern war nicht möglich, da beide ebenfalls hospitalisiert und nicht aufklärungsfähig waren. Das zuständige Familiengericht hat ein Pausieren des Sorgerechts beschlossen und der Transplantation zugestimmt.

## Organangebot

**Abb. 1:**
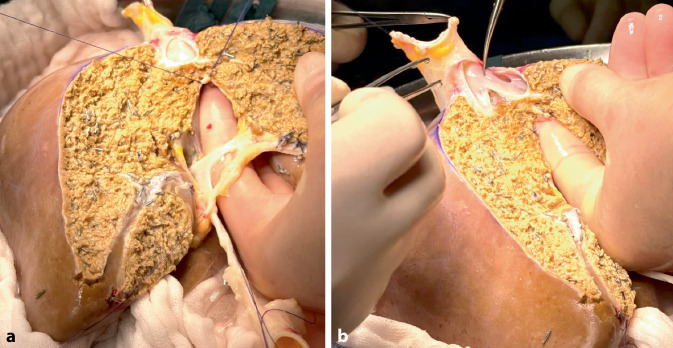
Gesplittetes Organ mit deutlicher makroskopischer Verfettung. **a** Ansicht auf die Resektionsfläche der Leber mit deutlicher Verfettung, **b** Ansicht auf die Teilung der Lebervenen bei dem „Splitten“ der Leber

An diesem Tag wurde ein rechts-erweitertes Split-Organ einer 36-jährigen Spenderin mit 165 cm Körpergröße, 75 kgKG, BMI 28, akzeptiert. Todesursache war eine intrazerebrale Blutung. Das Organ wurde für den links-lateralen Split in einem anderen Zentrum akzeptiert und dort gesplittet. Der verantwortliche Chirurg in dem anderen Zentrum informierte uns nach Sichtung des Organs über die unerwartete, hochgradige makroskopische Verfettung. Aufgrund des kritischen Zustands seines Empfängers würde er trotzdem dieses suboptimale Organ splitten (Abb. 1). Bei Ankunft des Organs betrug die kalte Ischämiezeit bereits 9 h 35 min.

## „Würden Sie diese Leber transplantieren?“

### Lösung.

Auch wir behielten die Akzeptanz des Organs bei und entschlossen uns, das Split-Organ an die Dual -HOPE-Maschinenperfusion anzuschließen. Zur Stabilisierung des Patienten und zur Zeitgewinnung bei zunehmender Enzephalopathie planten wir, die Plasmapherese intraoperativ fortzuführen.

## Hintergrund und Problematik

Die Transplantation solider Organe in Deutschland ist nach wie vor von erheblichen Hindernissen geprägt. Ein relativ geringes Organspendeaufkommen führt dazu, dass auch Organe minderer Qualität angenommen und transplantiert werden müssen.

Die Definition eines „marginalen Organs“ ist schwer, da dies von verschiedenen Faktoren abhängig ist. Zunächst gibt es den Organspender an sich, bei dem Alter, Komorbiditäten, BMI, Laborparameter und Dauer einer möglichen Reanimation die Qualität der Spenderorgane beeinflussen können. Die Qualität des Organs kann mittels histologischer Untersuchung in Bezug auf mikro- oder makrovesikuläre Verfettung, Fibrosegrad, Entzündung oder Nekrosen weiter beurteilt werden. Zu diesen vorbestehenden Faktoren kommt die Dauer oder die zu erwartende Dauer der kalten Ischämiezeit. Man geht davon aus, dass der Ischämiereperfusionsschaden des Organs von diesen Faktoren beeinflusst werden kann.

## Perfusionssysteme

Maschinenperfusionssysteme wurden entwickelt, um Organe möglichst physiologisch zu behandeln. Daher gibt es unterschiedliche Perfusionssysteme zur Auswahl. Man unterscheidet grob normotherme von hypothermer Maschinenperfusion, wobei die normothermen Systeme (NMP) zwingend mit Sauerstoffträgern, also Blut, perfundiert werden müssen. Bei den hypothermen Systemen kann man entweder nur die Pfortader perfundieren (HOPE: „hypothermic oxygenated perfusion“) oder auch die Arterie perfundieren (Dual HOPE) [1]. Allen Systemen wird Sauerstoff hinzugefügt.

## Welche Organe sollten perfundiert werden?

Es gibt einige Richtwerte, wann ein Organ von einer Maschinenperfusion profitieren kann. Zudem hat die Bundesärztekammer in ihren Richtlinien erweiterte Spenderkriterien festgelegt, anlehnend an diese legen manche Kliniken ihre Kriterien der Maschinenperfusion fest (Tab. 1).

**Tab. 1:** Erweiterte Spenderkriterien nach der aktuellen Definition der Bundesärztekammer und Verteilung im untersuchten Kollektiv (*n* = 291)

Erweiterte Spenderkriterien	Anzahl (%)
Alter des Spenders > 65 Jahre	30 (10,3)
Intensivtherapie einschließlich Beatmung des Spenders > 7 Tage	66 (22,7)
BMI des Spenders > 30	47 (16,2)
Steatosis hepatis (histologisch gesichert) > 40 %	22 (7,6)
SGOT oder SGPT > 3 × normal (letzter Wert vor der Spendermeldung)	60 (20,6)
Serumbilirubin > 3 mg/dl (letzter Wert vor der Spendermeldung)	229 (78,7)
Serumnatrium > 165 mmol/l (letzter Wert vor der Spendermeldung)	10 (3,4)

*BMI* Body-Mass-Index, *SGOT* Serum-Glutamat-Oxalacetat-Transaminase, *SGPT* Serum-Glutamat-Pyruvat-Transaminase

So hat unsere Institution ein Abkommen mit den Krankenkassen zur Vergütung der Maschinenperfusion unter folgenden Kriterien festgelegt (Abb. 3). Unabhängig von einer Kostenerstattung entscheiden wir zusätzlich bei ungünstigen Umständen oder sehr langer kalter Ischämiezeit, ob wir auch Organe perfundieren, bei denen die oben genannten Kriterien nicht erfüllt sind. Bis zu diesem Zeitpunkt wurden an unserer Institution nur Vollorgane an die Dual-HOPE-Maschinenperfusion angeschlossen.

## Präparation des Organs

**Abb. 2:**
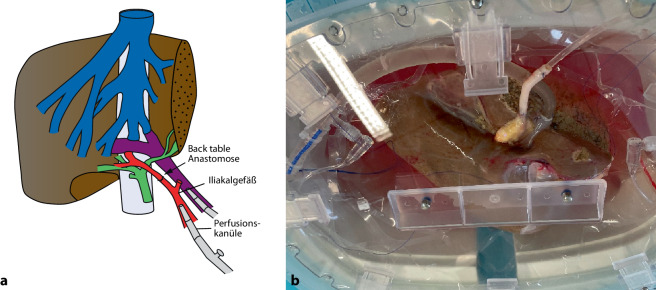
„Back table“ verlängerte Arterie zur Kanülierung, Organ an der Perfusion. **a** „Back table“-Verlängerung der Arterie zur Kanülierung mit einem Iliakalgefäß des Spenders, **b** Organ an der Perfusionmaschine

Um eine Kanülierung zu ermöglichen, musste die kurze rechte Arterie „back table“ mit einem Iliakalgefäß des Spenders verlängert werden. Diese Anastomose wurde mit 7/0 PDS durchgeführt (Abb. 2).

### Infobox Kriterien Leberperfusionsverfahren

Spenderkriterien gemäß aktuellem Studienprotokoll und Kriterien der Bundesärztekammer gemäß den gültigen Richtlinien für Spenden mit erweiterten Kriterien

- Alter > 65 Jahre
- oder Beatmung auf ICU > 7 Tage
- oder BMI > 30
- oder Natrium > 165 mol/l (letzter Wert vor Organangebot)
- oder Bilirubin gesamt > 3 mg/dl (letzter Wert vor Organangebot)
- oder GOT (AST), GPT (ALT) > 90 U/l (letzter Wert vor Organangebot)
- oder Fettleber (histologisch gesichert) > 40 %

Empfängerkriterien (müssen alle erfüllt sein)

- Alter > 18 Jahre
- Kein „high urgency“ (HU)

Im Fall von Leberretransplantationen oder kombinierten Lebertransplantationen kommt es durch den deutlich höheren Aufwand zu längeren Kaltischämiezeiten, die das Risikoprofil und die Organeignung gefährden. Diese Risiken werden durch die Maschinenperfusion deutlich minimiert. Der Einsatz der Maschinenperfusion ist daher bei Retransplantationen und kombinierten *Lebertransplantationen angezeigt.*

## Operationstechnik

Parallel zur „Back table“-Präparation wurde der Patient eingeleitet und die Hepatektomie vorbereitet. Um eine Perfusion von 2 h zu erreichen, verzögerten wir die Hepatektomie um ca. 45 min. Danach transplantierten wir den rechts-erweiterten Split in Piggy-back-Technik für die venöse Anastomose (PDS 5/0), Pfortader End zu End (PDS 6/0) und Arterie ebenfalls End zu End mit einem Teil des bereits vorher anastomosierten Iliakalgefäßes auf die A. hepatica propria (PDS 7/0) in Einzelknopftechnik. Der Gallengang wurde End zu End mit PDS 6/0 in Einzelknopftechnik anastomosiert. Der Absetzungsrand des linken Gallengangs wurde fortlaufend mit PDS 6/0 übernäht, ebenso die hiläre Platte.

## Operationsergebnis

**Abb. 3:**
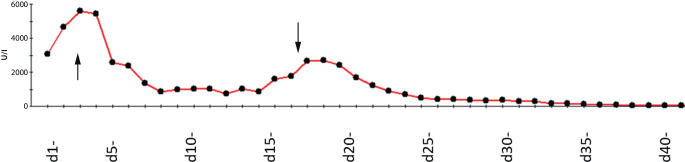
GOT-Werte im Verlauf in U/l, erster Peak akutes Leberversagen, zweiter Peak postoperativ

Die kalte Ischämiezeit betrug 12 h 30 min, die warme Ischämiezeit 33 min. Intraoperative Substitution betrug 1 EK, 140 ml Autotransfusion über den Cell-Saver. Maximaler Katecholaminbedarf intraoperativ betrug 0,7 µg/kg/min, der Laktatwert erreichte einen Peak von 7,2 mmol/l. Bei Übergabe auf die Intensivstation betrug der Katecholaminbedarf 0,35 µg/kg/min. Der Patient wurde am ersten postoperativen Tag extubiert ohne weiteren Katecholaminbedarf. Die Transaminasen stiegen bis auf GOT 2711 U/l und GPT 1888 U/l an und fielen dann zeitgerecht ab (Abb. 3).

## Postoperativer Verlauf

**Abb. 4:**
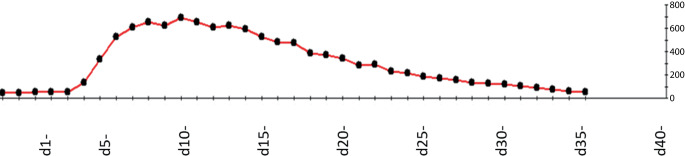
GGT-Werte im Verlauf in U/l

Am 5. postoperativen Tag kam es zu einem GGT-Anstieg und erhöhten Temperaturen. Die Antibiose wurde erweitert und ein Steroidstoß zur Behandlung einer potenziellen Abstoßung verabreicht, da eine Leberbiopsie einen erneuten Gerichtsbeschluss erforderlich gemacht hätte (Eltern selbst hospitalisiert). Unter Besserung der Laborparameter wurde der Patient am 9. postoperativen Tag auf die Normalstation verlegt (Abb. 4).

**Abb. 5:**
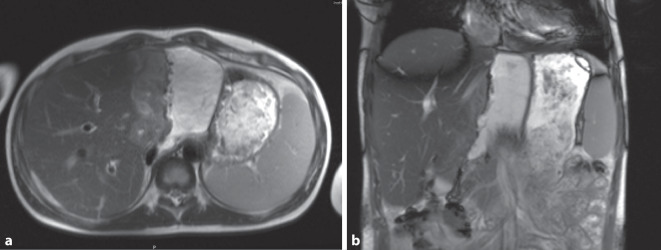
MRT Abdomen mit Biliom. **a** Biliom im MRT neben der Resektionsfläche, **b** Biliom in der Coronaransicht

Im weiteren Verlauf kam es zu einem erneuten Anstieg der Entzündungsparameter. In einem daraufhin durchgeführten MRT am 14. postoperativen Tag zeigte sich ein Flüssigkeitsverhalt an der Resektionsfläche, welcher einem infizierten Biliom entsprach (Abb. 5).

Der Verhalt wurde drainiert und mikrobiologisch untersucht. Aufgrund einer noch bestehenden Antibiose blieb die mikrobiologische Untersuchung ohne Keimnachweis. Eine darauffolgende ERCP zeigte eine Galleleckage, welche mittels 7‑French-Endoprothese überbrückt wurde. Kurz danach konnte der zuvor eingelegte Pigtail-Katheter entfernt werden. Am 26. postoperativen Tag wurde die i.v.-Antibiose mit Meronem und Vancomycin auf orales Amoxicillin und Clavulansäure umgestellt. Der Patient konnte nach weiterer Mobilisation und Kostaufbau sowie Klärung der häuslichen Versorgung am 32. postoperativen Tag in gutem Allgemeinzustand nach Hause entlassen werden.

## Welchen positiven Effekt hat die Maschinenperfusion?

Die Faktoren Steatose, Ischämiezeit und Parenchymdissektion akkumulieren zu einem deutlich erhöhten Zellschaden und Ischämie der Mitochondrien, dies führt zu einem Ischämiereperfusionsschaden [2]. Während der Ischämie produzieren Mitochondrien kein ATP, sondern ROS („reactive oxygen species“) [3, 4]. Diese werden dann bei der Reperfusion freigesetzt und bewirken eine mitochondrielle und mikrovaskuläre Dysfunktion, Schwellung des Organs, verlangsamte ATP-Reproduktion, Zytokinausschüttung und Inflammation [3]. Vergleichende Studien haben gezeigt, dass sowohl die normotherme Maschinenperfusion (NMP) als auch HOPE die Primärfunktion des Organs positiv beeinflussen, jedoch HOPE die Mitochondrien durch eine kalte Reoxygenierung besser schützt [5]. Dieser protektive Effekt auf die Mitochondrien soll auch das angeborene Immunsystem herunterregulieren und somit die Rate an akuten Abstoßungen reduzieren [6].

## War unsere Entscheidung richtig?

Trotz optimaler Therapie unseres Empfängers verschlechterte sich sein klinischer Zustand mit nicht ganz einzuschätzender Dynamik. Nach Erhalt des nach Spenderdaten sehr guten Organangebots akzeptierten wir dieses unter Vorbehalt und der Rücksprache mit dem anderen Zentrum. Als dieses sich wider Erwarten als makroskopisch deutlich steatotisch darstellte, behielten wir aufgrund des Zustands des Patienten die Akzeptanz bei. Bei Ankunft des Organs bestand dann schon eine deutlich verlängerte kalte Ischämiezeit. Somit sprachen nun mehrere Faktoren dafür, dieses Organ an die Dual-HOPE- Perfusion anzuschließen, obwohl die offiziellen Kriterien nicht erfüllt waren.

## Erfahrungen mit Split-Leber-Maschinenperfusion

Aufgrund des schmalen Gefäßlumens der Arterie des rechts-erweiterten Splits verlängerten wir diese mit einem Iliakalgefäß des Spenders (Abb. 2). In der Literatur gibt es nur wenige Daten über Split-Leber-Maschinenperfusion. Einige Zentren haben Fallserien von Split-Prozeduren an der HOPE Maschinenperfusion beschrieben [7, 8]. Zur Vermeidung von arteriellen Komplikationen wurden diese 3 Split-Prozeduren nur mit einer Pfortaderkanülierung durchgeführt. Rossignol et al. [9] haben insgesamt 8 pädiatrische Lebertransplantation nach Split-Prozeduren an HOPE beschrieben und die Split-Organe dann über einen medianen Zeitraum von 125 min weiter perfundiert. In allen Fällen wurde eine sofortige Organfunktion beschrieben und ein deutlich reduziertes Reperfusionssyndrom im Vergleich zu Split-Lebertransplantation nach kalter Lagerung. Auch in unserem Fall kam es zu keinem wesentlichen Reperfusionssyndrom und primärer Organfunktionsaufnahme. Ob eine arterielle Perfusion notwendig ist, müssten weiterführende Studien untersuchen.

## Maschinenperfusion und biliäre Komplikationen

Schlegel und Dutkowski zeigten, dass die HOPE-Maschinenperfusion biliäre Komplikationen senken kann [10]. Sie fassten Ergebnisse von insgesamt 9 Studien in den letzten 5 Jahren zusammen. Insbesondere wurde hier auch auf die spezifischen Effekte der Ischämiereperfusion auf Gallengangsepithelien auf zellulärer Ebene eingegangen. Unser Patient entwickelte eine Galleleckage mit Biliom und konsekutiver Stentung trotz der HOPE-Perfusion. Wie sich die Gallenwegskomplikation ohne HOPE-Perfusion entwickelt hätte, bleibt natürlich spekulativ.

## Fazit

Zusammenfassend muss erneut infrage gestellt werden, ob die derzeit geltenden Richtlinien zur Allokation von Split-Lebern in Deutschland nicht die Qualität dieser partiellen Grafts gefährdet. In-situ-Splits würden die Ischämiezeit beider Grafts deutlich reduzieren und einander angleichen. Ebenso würde die Transplantation beider Teile an einem Zentrum die Bedingungen für den zweiten Graft deutlich verbessern. Unter den gegebenen Umständen wäre es sicherlich sinnvoll, die Split-Prozedur, wenn möglich, an einer HOPE-Perfusion durchzuführen.
